# Intra-abdominal fat volume estimation by multi-detector rows computed tomography: relevance in surgical fellowship training program in Shanghai: a retrospective study

**DOI:** 10.7717/peerj.15156

**Published:** 2023-04-19

**Authors:** Jenifei Shah, Suyue Yu, Jingyi Huang, Lu Zang, Tian Li, Zhenglun Zhu

**Affiliations:** 1 Department of General Surgery, Ruijin Hospital, Shanghai Jiao Tong University School of Medicine, Shanghai, China; 2 Department of Urology, Ruijin Hospital, Shanghai Jiao Tong University School of Medicine, Shanghai, China; 3 School of Basci Med, Fouth Military Medical University, Xi’an, China

**Keywords:** Gastric cancer, D2 gastrectomy, Intra-abdominal fat volume, Blood loss, Obesity, Fellowship training

## Abstract

**Background:**

Intra-abdominal fat volume (IFV) has been shown to have a negative impact on surgical outcomes in gastric cancer (GC) and other gastrointestinal surgeries. The purpose of this study is to look into the relationship between IFV and perioperative outcomes in GC patients using multi-detector rows computed tomography (MDCT) and assess the importance of implementing this observation in current surgical fellowship training programs.

**Methods:**

Patients with GC who underwent open D2 gastrectomy between May 2015 and September 2017 were included in the study. Based on MDCT estimation, patients were divided into high IFV (IFV ≥ 3,000 ml) and low IFV (IFV < 3,000 ml) groups. Perioperative outcomes for cancer staging, type of gastrectomy, intraoperative blood loss (IBL), anastomotic leakage, and hospital stay were compared between the two groups. This study was registered as CTR2200059886.

**Results:**

Out of 226 patients, 54 had early gastric carcinoma (EGC), while 172 had advanced gastric carcinoma (AGC). There were 64 patients in the high IFV group and 162 in the low IFV group. The high IFV group had significantly higher IBL mean values (*p* = 0.008). Therefore, having a high IFV was a risk factor for the occurrence of perioperative complications (*p* = 0.008).

**Conclusions:**

High IFV estimated by MDCT prior to GC surgery was associated with increased IBL and postoperative complications. Incorporating this CT-IFV estimation into surgical fellowship programs may aid aspiring surgeons in selecting patients during independent practice in their learning curve and surgical practice for the most appropriate approach for treating GC patients.

## Introduction

Medical education is a lifelong continuum. In 2010, Shanghai led the way in implementing a standardized residency training program (SRT), which expanded to 44 hospitals by 2013 and is now a requirement for employment in the city (Yang-Huang et al., 2019; Zhang & Fang, 2013). China revised its medical education system gradually with the implementation of higher education reform in 1998 and health reform in 2009 (Zhu, Li & Chen, 2016). The fellowship specialty training programs are favored by trainees (Yang-Huang et al., 2019), and a 2019 survey of SRT residents highlighted the importance of a supportive environment for better job satisfaction (Zhang et al., 2021). A systematic review demonstrates that centers with fellowship programs have better patient outcomes (Johnston et al., 2015).

Obesity is associated with an increased risk of complications following abdominal surgery (Moon et al., 2008; Abu-Abid, Szold & Klausner, 2002). Visceral fat estimated as intra-abdominal fat volume (IFV) using multi-detector rows computed tomography (MDCT) (Lv, Beeharry & Zhu, 2019; Moon et al., 2008) is reportedly a more accurate indicator of visceral fat than the body mass index (BMI) (Yoshikawa et al., 2011; Zhu & Yan, 2011). Gastrectomy with D2 lymphadenectomy is a commonly performed procedure in China for gastric cancer (GC)  (Japanese Gastric Cancer Association, 2021; Bonenkamp et al., 1999; Price et al., 2006). CT-IFV estimation before surgery may be an additional armament in selection and evaluation of patients prior to gastrectomy or other abdominal surgeries (Liu et al., 2018; Yamada et al., 2008; Taniguchi et al., 2021).

Incorporating CT-IFV estimation to evaluate patients will add value to SRT programs and help the trainee surgeons during their learning curve to optimize perioperative outcomes of gastrectomy and other abdominal surgeries.

## Patients and Methods

### Patients

From May 2015 to September 2017, patients with gastric carcinoma who underwent open gastrectomy with D2 lymphadenectomy in the Department of General Surgery of Ruijin Hospital affiliated to Shanghai Jiao Tong University School of Medicine were included in this study. This retrospective study was registered as CTR2200059886 and approved by the Ruijin Hospital Ethics Committee, Shanghai Jiao Tong University School of Medicine, China (2018-151).

**Figure 1:**
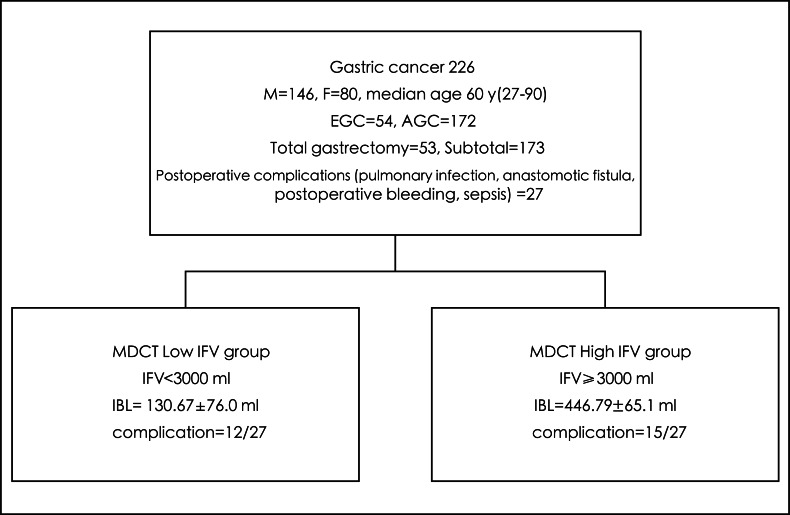
Flow chart of patient recruitment and outcome of gastric surgery in high *vs* low intra-abdominal fat volume (IFV) measured by multi-detector rows computed tomography (MDCT).

The same surgical fellowship trainee and team operated on the patients according to the hospital’s protocol. Another experienced physician was responsible for dissecting and inspecting the specimen’s lymph nodes prior to sending them to the pathology department. The operation time, measured from skin incision to skin suturing, was noted. The volume of intraoperative blood loss (IBL) was recorded according to the collection canister readings and the number of gauzes used (Fig. 1).

### CT-IFV estimation

The MDCT was performed using a 4-detector row CT scanner in patients who had fasted for at least 8 h. Patients were asked to maintain a supine position, and the localization image was first scanned using the umbilicus as a central reference point, with the scan ranging from the upper edge of the right diaphragm to the pubic symphysis. After an unenhanced scan of the upper abdomen, a 100 ml bolus of nonionic iodine contrast agent (Ultravist; Schering, Germany) was injected into the antecubital vein at a flow rate of 3 ml/s *via* a 20-gauge needle using an automatic injector. Then, an 80 ml bolus of contrast agent was injected at a 1 ml/s flow rate. CT acquisitions were conducted in three different phases: arterial phase (start delay of 30 s), portal venous phase (start delay of 75 s), and equilibrium phase (start delay of 180 s). The parameters were as follows: slice 10 mm; interlayer spacing 10 mm; pitch 1.375:1; 100 kV; 200 mA. Upon completion of the scan, original images with slices of 10 mm were thinned out to 1.25 mm before their transfer to Research Frontier, Syngo Via, Version VB20, Siemens Healthineer for reorganization.

**Figure 2:**
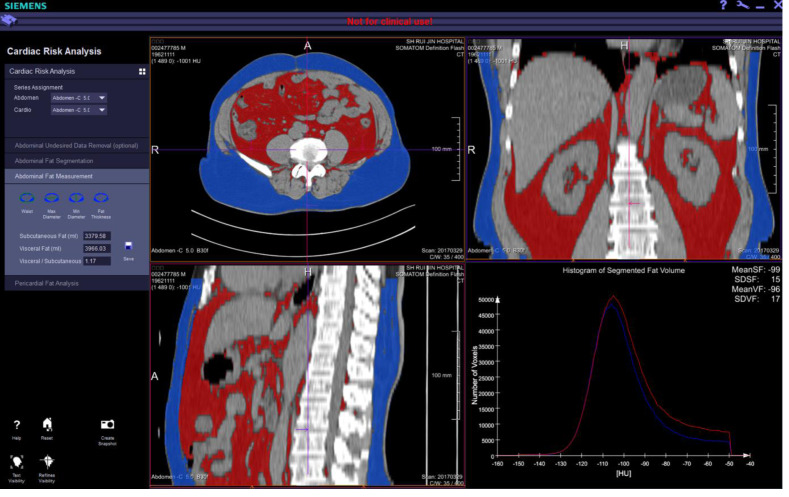
The intra-abdominal fat volume (IFV) measured by multi-detector rows computed tomography (MDCT) obtained on comprehensive view at different levels in coronal, sagittal and cross-sectional images with multiplanar reconstruction (MPR) images.

The CT images were transferred to the work platform in the digital imaging and communications in medicine (DICOM) format. The CT units (in Hounsfield units) from −250 ± 3 to −50 ± 3 Hu was defined as adipose tissue. Histogram software (Research Frontier, Syngo Via, Version VB20, Siemens Healthineer) was used to plot a histogram of tissue volume. A histogram of segmented fat volume was computed by focusing on the tissue with CT numbers from −250 ± 3 to −50 ± 3 Hu. The peak of this curve represented the range of CT numbers with the largest proportion of fat tissue. The colored portion in the reconstructed image, which indicated the abdominal fat tissue, was used to calculate the intra-abdominal fat volume (IFV). The distribution of abdominal fat at different levels was comprehensively determined using the coronal, sagittal and cross-sectional images with multiplanar reconstruction (MPR) (Fig. 2).

### Variables

Since the median IFV in the patients was 3,000 ml, this value was used to stratify patients into two groups: high IFV group (IFV ≥ 3,000 ml) and low IFV group (IFV < 3,000 ml). Other variables such as intraoperative blood loss (IBL), operation time, postoperative hospital time, number of dissected lymph nodes and early postoperative complications were also compared between the two groups.

### Statistical analysis

Descriptive statistics were performed to illustrate the baseline characteristics. Chi-squared test and *t*-test were used to compare the different variables between the two groups, which included: age, gender, surgical method, stage of cancer, operation time, the volume of blood loss and operative complications. Wilcoxon rank-sum test was applied to compare the number of dissected lymph nodes.

Statistical analyses were carried out using SAS ‘PROC GLM’ 8.0 (SAS Institute, Cary, NC, USA). A 2-tailed *p*-value ≤0.05 was considered statistically significant.

## Results

There were 226 GC patients, 146 males and 80 females, with a median age of 60 years (ranging from 27 to 90 years); 54 had early EGC, and 172 had AGC. A total of 173 patients underwent distal subtotal gastrectomy with D2 lymphadenectomy, while 53 underwent total gastrectomy with D2 lymphadenectomy. None of the patients had received adjuvant chemoradiotherapy.

IBL was significantly higher in the high IFV group compared to the low IFV group, 446.79 ± 65.1 ml *vs.* 130.67 ± 76.0 ml, *p* = 0.008.

There were no significant differences between the high and low IFV groups for other variables like gender (*p* = 0.259), surgical method (*p* = 0.151) or GC stage (*p* = 0.428), average age 63.66 ± 7.9 years *vs.* 61.19 ± 9.9 years (*p* = 0.644), operation time 240.55 ± 63.7 min *vs.* 237.92 ± 70.3 (*p* = 0.798) and median number of dissected lymph nodes 23 (ranging from 15 to 40) *vs.* 24 (ranging from 13 to 78) (Table 1).

There were 27 cases (12.3%) of postoperative complications; seven patients had pulmonary infections (3.1%), seven patients had anastomotic fistula (3.1%), six patients had postoperative bleeding (2.7%), and five patients had sepsis (2.2%) (Table 2). Among the 27 patients that had complications, 15 were in high IFV patients compared to 12 in the low IFV group (*p* = 0.008) (Tables 2 and 3).

## Discussion

In the present study, the multivariate analysis revealed that high IFV patients had more blood loss during surgery (*p* = 0.008). This might be related to more extensive fat and vascular supply destruction, resulting in increased intraoperative bleeding in high IFV patients  (Makino et al., 2008).

Our findings show a higher incidence of overall complications in patients with high IFV in contrast to patients with low IFV, 15 *vs.* 12 (*p* = 0.008). Similarly, previous studies have shown that patients with a larger amount of visceral fat had a higher risk of early postoperative complications (Tokunaga et al., 2009; Inagawa et al., 2000). The most common postoperative complication is intra-abdominal infection (9.6%) followed by anastomotic leakage (4.4%), especially in patients undergoing gastrectomy with Roux-en-Y anastomosis. This could be because performing esophagojejunal anastomosis becomes more difficult in patients with a large amount of abdominal fat covering the mesentery and omentum. Taken together, these findings may reflect worse operability in patients with a high IFV compared to those with low IFV.

**Table 1:** Association between Intra-abdominal fat volume (IFV) and age, gender, surgical method, peri-operative outcome of gastrectomy for gastric cancer.

	High IFV (*n* = 64)	Low IFV (*n* = 162)	*p* value
Age (*y*)	63.66 ± 7.9	62.60 ± 10.1	0.644
Gender			0.259
Male	45	101	
Female	19	61	
Surgical Method			0.151
Total Gastrectomy	14	39	
Subtotal Gastrectomy	50	123	
Stage			0.4275
Early Gastric Cancer	13	41	
Advanced Gastric Cancer	51	121	
Dissected Lymph Nodes (*n*)	23 (15–40)	24 (13–78)	0.376
Operation Time (min)	240.55 ± 63.7	237.92 ± 70.3	0.798
Volume of blood loss (ml)	446.79 ± 65.1	130.67 ± 76.0	0.008

**Table 2:** Postoperative complications of gastric cancer in patients who underwent D2 gastrectomy.

	Total	Male	Female
Pulmonary Infection	7 (3.1%)	6	1
Postoperative Bleeding	6 (2.7%)	4	2
Incision Infection	1 (0.44%)	0	1
Anastomotic Fistula	7 (3.1%)	4	3
Sepsis	5 (2.2%)	4	1
Deep Vein Thrombosis	1 (0.44%)	0	1
Total	27 (12.3%)	18	9

**Table 3:** Association between IFV and postoperative complications of gastric cancer following D2 gastrectomy.

	Postoperative complication		X2 value	*P* value
	Yes (*n* = 27)	No (*n* = 199)		
IFV			11.2	0.008
High IFV	15	49		
Low IFV	12	150		

The BMI is a widely used and accepted indicator of obesity (World Health Organization, 2021). General obesity is more common in Western countries, while visceral obesity is predominant in China and the Asian population (Sjöström et al., 1986). However, BMI does not accurately reflect the effect of visceral obesity on the outcome of gastric cancer surgery (Zhu & Yan, 2011). Reports have shown that high IFV patients and obese patients undergoing extensive abdominal surgery have more complications, longer hospital stay and higher medical costs compared to low IFV patients (Makino et al., 2008; Tokunaga et al., 2009).

Fellowship training, built on the foundation of residency training, is a proven method for developing independent specialists capable of performing standardized diagnosis and treatment of the diseases in their fields of study. China revised its medical education system with the implementation of higher education reform in 1998 and health reform in 2009 (Zhu, Li & Chen, 2016). In 2010, Shanghai took the initiative in developing and implementing a standardized residency training (SRT) program that is now required for employment in the city (Yang-Huang et al., 2019; Zhang & Fang, 2013). Residents have the option of selecting a training hospital and must pass hospital-level entrance examinations prior to enrollment. SRT allows for a customizable program to accommodate graduates with varying educational and clinical experience, including those with bachelor’s, master’s, and doctoral degrees, by allowing them to complete three, two, or one year of residency training, respectively. The SRT incorporates sub-specialty training paths that residents can choose from with different weighted credits according to the departments to prepare them for their specialization. Although fellowship specialty training programs are preferred (Yang-Huang et al., 2019), it also requires proper consideration from hospital management to provide a supportive work environment that addresses the residents’ exhausting schedule and anxiety, as highlighted in a 2019 survey of SRT residents at Guizhou Provincial People’s Hospital, China  (Zhang et al., 2021).

For a specialist surgeon, strong emphasis should be placed on honing specialized surgical techniques. A specialist surgeon is expected to perform more complex surgical procedures and be capable of operating independently, which is one of the important criteria used to assess the ability of a general surgeon. In this study, IFV was accurately measured using MDCT and was associated with a better clinical outcome in patients with lower IFV. This may serve as an additional reference for aspiring specialists during their fellowship training program, assisting them in performing a more thorough patient evaluation and planning for the necessary surgical procedures.

Obesity has been on the rise in recent years, partly as a result of rising living standards and westernization of the Chinese population’s diet, becoming one of the leading causes of chronic diseases such as hypertension, diabetes, cardiovascular disease, and colon cancer (Abu-Abid, Szold & Klausner, 2002). Studies have previously shown that the Chinese visceral adiposity index (CVAI) is a predictor for Type-2 diabetes mellitus and hypertension  (Han et al., 2021a; Han et al., 2021b).

The development of MDCT has made it possible to measure adipose tissue volume and estimate the amount and distribution of visceral fat (Makino et al., 2008). The MDCT has several advantages, including accurate localization and quantification, repeatability, and high-density resolution, thus reducing the time taken to measure and improving accuracy without requiring additional testing or expense.

In general, the upper abdomen has a concentrated area of fat tissue. Excess visceral fat has previously been considered an accurate predictor for operability and surgical complications (Moon et al., 2008; Lv, Beeharry & Zhu, 2019; Yamada et al., 2008; Taniguchi et al., 2021).

Studies have shown an increased incidence of postoperative pancreatic fistula in patients with a larger amount of intra-abdominal fat (Tanaka et al., 2009). This might be due to the increased fragility of intra-abdominal fat and difficulty in exposing the pancreas during surgery. This added difficulty in distinguishing between healthy pancreatic tissue and peripancreatic fat may further increase the risk of damaging normal pancreatic tissue. Studies have demonstrated a possible correlation between the difficulty of surgery and the distribution of abdominal fat  (Yamada et al., 2008; Taniguchi et al., 2021; Seki et al., 2007; Tsujinaka et al., 2008). These findings underscore the importance of a targeted, stepwise training program to train future doctors in patient selection and treatment strategy decisions to minimize complications and improve the overall surgical outcome of GC patients. In this study, we found that there were significantly more IBL and complications in patients with high IFV compared to patients in the low IFV group.

In patients with high IFV, the synthesis and release of adipokines, such as TNF-a, leptin, lipocalin, interleukin-6, and angiotensin were significantly increased. Interestingly, the occurrence of postoperative inflammatory response syndrome in gastric cancer patients is closely related to the excessive release of adipokines (Tsujinaka et al., 2007; Bastard et al., 2006). The mode and location of fat storage vary between populations or individuals; for example, Asian men tend to store fat predominantly in the abdominal cavity, whereas Asian women store fat predominantly in the lower abdomen, buttocks, and thighs (Nauli & Matin, 2019).

Our findings revealed a significant correlation between increased complication rates in GC patients undergoing surgery and technical difficulties associated with operating on high IFV patients and obese patients. This is critical to keep in mind during the postgraduate surgery fellowship program for gastrointestinal surgery, particularly for upper abdomen surgery involving the stomach and surrounding organs. Along with the extent of surgery, aspiring doctors should take into consideration the IFV status and obesity while selecting patients for surgery as part of the learning curve of the surgery fellowship training program. Importantly, IFV and obesity are important factors that should warrant more attention from surgeons to minimize postoperative complications and mortality.

## Conclusion

This study shows that the high intra-abdominal fat volume (IFV) measured by multi-detector rows computed tomography (MDCT) in gastric cancer patients had higher intraoperative blood loss and postoperative complications than low IFV patients. The IFV-CT protocol may raise awareness and prepare the surgical trainees for the possible difficulties during surgery in order to minimize high IFV-related postoperative complications and mortality.

##  Supplemental Information

10.7717/peerj.15156/supp-1 Data S1 Raw data of patients

## Abbreviations

IFV: Intra-abdominal fat volume
GC: Gastric cancer
MDCT: Multi-detector rows computed tomography
IBL: Intraoperative blood loss
EGC: Early gastric carcinoma
AGC: Advanced gastric carcinoma
SRT: Standardized residency training program
BMI: Body mass index
DICOM: Digital imaging and communications in medicine
MPR: Multiplanar reconstruction
CVAI: Chinese visceral adiposity index

## Additional Information and Declarations

### Data Availability

The following information was supplied regarding data availability: The raw data is available in the Supplemental File and at Zenodo: Tian Li. (2023). Intra-abdominal fat volume estimation by multi-detector rows computed tomography: relevance in surgical fellowship training program in Shanghai: a retrospective study https://doi.org/10.5281/zenodo.7712873.

## References

[ref-1] Abu-Abid S, Szold A, Klausner J (2002). Obesity and cancer. Journal of Medicine.

[ref-2] Bastard JP, Maachi M, Lagathu C, Kim MJ, Caron M, Vidal H, Capeau J, Feve B (2006). Recent advances in the relationship between obesity, inflammation, and insulin resistance. European Cytokine Network.

[ref-3] Bonenkamp JJ, Hermans J, Sasako M, Van de Velde CJ, Welvaart K, Songun I, Dutch Gastric Cancer Group (1999). Extended lymph-node dissection for gastric cancer. The New England Journal of Medicine.

[ref-4] Han M, Qie R, Li Q, Liu L, Huang S, Wu X, Zhang D, Cheng C, Zhao Y, Liu D, Guo C, Zhou Q, Tian G, Zhang Y, Wu Y, Li Y, Yang X, Zhao Y, Feng Y, Qin P, Hu F, Zhang M, Hu D (2021a). Chinese visceral adiposity index, a novel indicator of visceral obesity for assessing the risk of incident hypertension in a prospective cohort study. British Journal of Nutrition.

[ref-5] Han M, Qin P, Li Q, Qie R, Liu L, Zhao Y, Liu D, Zhang D, Guo C, Zhou Q, Tian G, Huang S, Wu X, Li Y, Yang X, Zhao Y, Feng Y, Liu Y, Li H, Sun X, Chen Q, Wang T, Chen X, Hu D, Zhang M (2021b). Chinese visceral adiposity index: a reliable indicator of visceral fat function associated with risk of type 2 diabetes. Diabetes/Metabolism Research and Reviews.

[ref-6] Inagawa S, Adachi S, Oda T, Kawamoto T, Koike N, Fukao K (2000). Effect of fat volume on postoperative complications and survival rate after D2 dissection for gastric cancer. International Gastric Cancer Association and the Japanese Gastric Cancer Association.

[ref-7] Japanese Gastric Cancer Association (2021). Japanese gastric cancer treatment guidelines 2018 (5th edition). Gastric Cancer.

[ref-8] Johnston MJ, Singh P, Pucher PH, Fitzgerald JEF, Aggarwal R, Arora S, Darzi A (2015). Systematic review with meta-analysis of the impact of surgical fellowship training on patient outcomes. British Journal of Surgery.

[ref-9] Liu Y, Guo D, Niu Z, Wang Y, Fu G, Zhou Y, Xue Q, Jin X, Gong Z (2018). Prediction of the risk of laparoscopy-assisted gastrectomy by comparing visceral fat area and body mass index. Gastroenterology Research and Practice.

[ref-10] Lv T, Beeharry MK, Zhu Z-L (2019). Impact of intra-peritoneal fat distribution on intra-operative bleeding volume with D2 lymphadenectomy in Chinese patients with gastric cancer. Asian Journal of Surgery.

[ref-11] Makino H, Kunisaki C, Akiyama H, Ono HA, Kosaka T, Takagawa R, Nagano Y, Fujii S, Shimada H (2008). Effect of obesity on intraoperative bleeding volume in open gastrectomy with D2 lymph-node dissection for gastric cancer. Patient Safety in Surgery.

[ref-12] Moon H-G, Ju Y-T, Jeong C-Y, Jung E-J, Lee Y-J, Hong S-C, Ha W-S, Park S-T, Choi S-K (2008). Visceral obesity may affect oncologic outcome in patients with colorectal cancer. Annals of Surgical Oncology.

[ref-13] Nauli AM, Matin S (2019). Why do men accumulate abdominal visceral fat?. Frontiers in Physiology.

[ref-14] Price GM, Uauy R, Breeze E, Bulpitt CJ, Fletcher AE (2006). Weight, shape, and mortality risk in older persons: elevated waist-hip ratio, not high body mass index, is associated with a greater risk of death. The American Journal of Clinical Nutrition.

[ref-15] Seki Y, Ohue M, Sekimoto M, Takiguchi S, Takemasa I, Ikeda M, Yamamoto H, Monden M (2007). Evaluation of the technical difficulty performing laparoscopic resection of a rectosigmoid carcinoma: visceral fat reflects technical difficulty more accurately than body mass index. Surgical Endoscopy.

[ref-16] Sjöström L, Kvist H, Cederblad A, Tylén U (1986). Determination of total adipose tissue and body fat in women by computed tomography, 40K, and tritium. American Journal of Physiology.

[ref-17] Tanaka K, Miyashiro I, Yano M, Kishi K, Motoori M, Seki Y, Noura S, Ohue M, Yamada T, Ohigashi H, Ishikawa O (2009). Accumulation of excess visceral fat is a risk factor for pancreatic fistula formation after total gastrectomy. Annals of Surgical Oncology.

[ref-18] Taniguchi Y, Kurokawa Y, Takahashi T, Saito T, Yamashita K, Tanaka K, Makino T, Yamasaki M, Nakajima K, Eguchi H, Doki Y (2021). Impacts of preoperative psoas muscle mass and visceral fat area on postoperative short- and long-term outcomes in patients with gastric cancer. World Journal of Surgery.

[ref-19] Tokunaga M, Hiki N, Fukunaga T, Ogura T, Miyata S, Yamaguchi T (2009). Effect of individual fat areas on early surgical outcomes after open gastrectomy for gastric cancer. British Journal of Surgery.

[ref-20] Tsujinaka S, Konishi F, Kawamura YJ, Saito M, Tajima N, Tanaka O, Lefor AT (2008). Visceral obesity predicts surgical outcomes after laparoscopic colectomy for sigmoid colon cancer. Diseases of the Colon & Rectum.

[ref-21] Tsujinaka T, Sasako M, Yamamoto S, Sano T, Kurokawa Y, Nashimoto A (2007). Influence of overweight on surgical complications for gastric cancer: results from a randomized control trial comparing D2 and extended para-aortic D3 lymphadenectomy (JCOG9501). Annals of Surgical Oncology.

[ref-22] World Health Organization Obesity and Overweight. https://www.who.int/en/news-room/fact-sheets/detail/obesity-and-overweight.

[ref-23] Yamada H, Kojima K, Inokuchi M, Kawano T, Sugihara K (2008). Effect of obesity on technical feasibility and postoperative outcomes of laparoscopy-assisted distal gastrectomy—comparison with open distal gastrectomy. Journal of Gastrointestinal Surgery.

[ref-24] Yang-Huang J, Qian W, Zhang K, Shi L, Huang J (2019). The influence of standardized residency training on trainees’ willingness to become a doctor: a comparison between traditional Chinese medicine and Western medicine. International Journal of Environmental Research and Public Health.

[ref-25] Yoshikawa K, Shimada M, Kurita N, Iwata T, Nishioka M, Morimoto S, Miyatani T, Komatsu M, Mikami T, Kashihara H (2011). Visceral fat area is superior to body mass index as a predictive factor for risk with laparoscopy-assisted gastrectomy for gastric cancer. Surgical Endoscopy.

[ref-26] Zhang H, Chen D, Cui N, Zou P, Shao J, Wang X, Zhang Y, Du J, Du C, Zhou G, Zheng D (2021). Explaining job satisfaction among residents in standardized residency training programs: a serial multiple mediation model. Risk Management and Healthcare Policy.

[ref-27] Zhang K, Fang L (2013). The standardized training of resident physicians boosting the new medical reform: based on the three years practice effect of Shanghai. China Health Insurance.

[ref-28] Zhu J, Li W, Chen L (2016). Doctors in China: improving quality through modernisation of residency education. The Lancet.

[ref-29] Zhu Z, Yan M (2011). Body mass index and waist circumference do not affect early surgical outcomes in gastric cancer patients with D2 lymphadenectomy. Journal of Surgery Concepts & Practice.

